# Paranasal Osteoma: The Importance of Surveillance

**DOI:** 10.7759/cureus.44696

**Published:** 2023-09-05

**Authors:** Guilherme Movio, Shadaba Ahmed

**Affiliations:** 1 Medical Education, Lancaster Medical School, London, GBR; 2 Otolaryngology, University Hospital Morecambe Bay Trust, Lancaster, GBR

**Keywords:** epiphora, nasal obstruction, paranasal osteoma, sinus, osteoma

## Abstract

Paranasal osteomas are rare benign bone tumours originating within the paranasal sinuses. Despite their benign nature, these slow-growing osseous lesions can lead to a spectrum of symptoms ranging from asymptomatic discovery to nasal obstruction, headache, facial deformity, and ophthalmological complications. We present the case of a 52-year-old female who initially presented with chronic sinusitis-like symptoms in 2008 and was incidentally found to have a small right-sided paranasal sinus osteoma on CT. Eleven years later, in 2019, she re-presented with new symptoms of unilateral nasal obstruction, epiphora, and restriction in her lateral gaze and was diagnosed with a large osteoma causing structural and ophthalmological issues (proptosis and epiphora). Endoscopic removal of the osteoma successfully alleviated her symptoms. This case emphasises the importance of surveillance of paranasal osteomas as there are no formal guidelines to support clinicians, as even though they grow slowly, they can eventually lead to significant "mass-effect" symptoms and impact local structures. Thus, monitoring and consideration of surgical intervention are crucial to managing these lesions effectively.

## Introduction

Paranasal osteomas are rare benign bone tumours originating within the paranasal sinuses. This pathology continues to captivate the interest of clinicians, radiologists, and researchers due to its intriguing clinical presentations, diverse morphologies, and evolving diagnostic and management strategies. These slow-growing osseous lesions arise from the craniofacial skeleton, primarily affecting the frontal, ethmoid, and maxillary sinuses [1-3]. It has been estimated that they grow at a mean rate of 1.61mm per year; however, some have been reported to grow much faster [4]. Their precise aetiology remains uncertain, encompassing theories involving genetic predisposition, developmental anomalies, and aberrations in osteoblastic activity due to trauma and inflammation [1,2]. Despite their benign nature, paranasal osteomas can lead to a spectrum of symptoms ranging from asymptomatic discovery to pathological manifestations such as nasal obstruction, headache, facial deformity, and ophthalmological complications [1].

We describe a patient who presented in 2008 and was consequently diagnosed with chronic sinusitis. She also underwent computerised tomography (CT) of the sinuses, which revealed an incidental small right-sided paranasal sinus osteoma. It was deemed an incidental finding as it was thought to not be contributing to her symptoms. Eleven years later, in 2019, she re-presented with unilateral nasal obstruction, epiphora, and restriction in her lateral gaze. She was referred to ears, nose, and throat (ENT) services, where a large osteoma was diagnosed on CT. She underwent endoscopic removal and was symptom-free at follow-up [2]. Our case emphasises that although osteomas are slow-growing, surveillance may prevent future comorbidity.

## Case presentation

**Figure 1:**
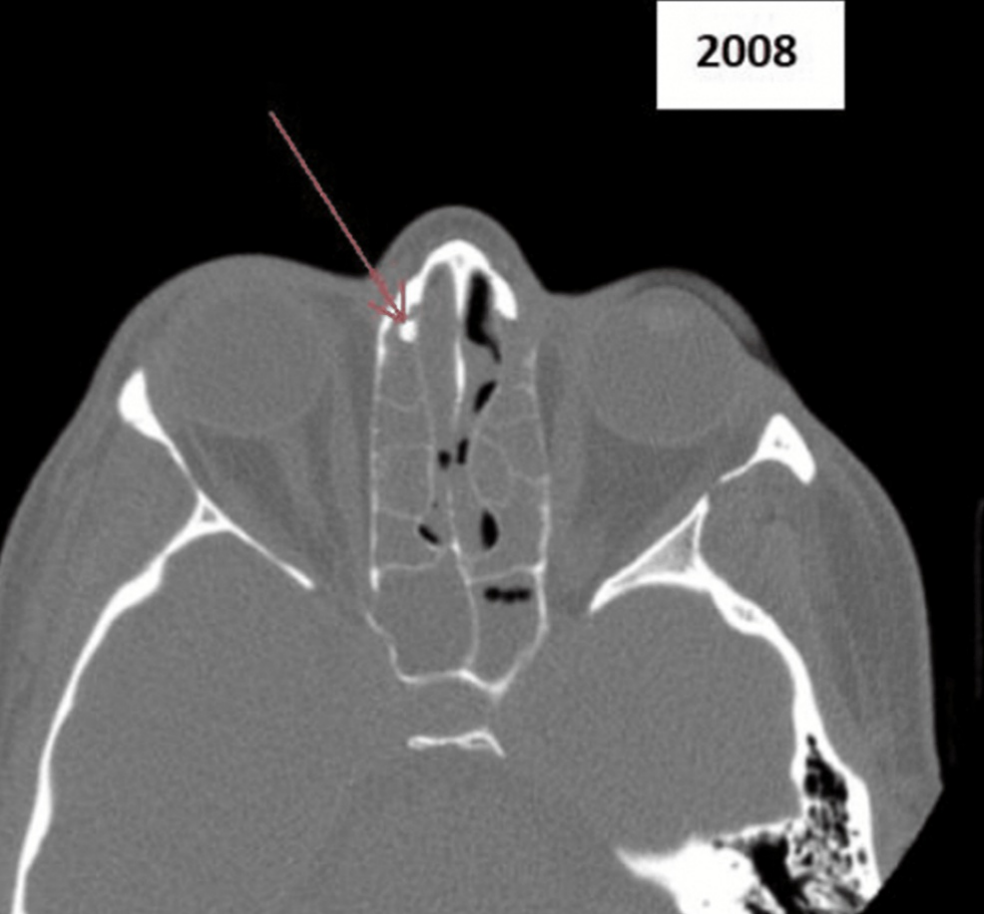
CT image of sinuses in 2008 The red arrow is showing the osteoma.

A 52-year-old female presented to her general practitioner (GP) with symptoms of chronic nasal obstruction, facial pain, headaches, and post-nasal drip in 2008. She had no relevant past medical history. A referral was made to the ENT services, where a CT of the sinuses was conducted, and she was diagnosed with chronic sinusitis. However, a small right-sided paranasal sinus osteoma was identified (Figure 1). This was not thought to be the cause of her presentation. She was therefore treated for chronic sinusitis and discharged. The osteoma was managed conservatively, however, there was no follow-up due to a lack of appropriate guidelines and supporting evidence. Her sinusitis symptoms resolved with intranasal steroids and regular irrigation.

**Figure 2:**
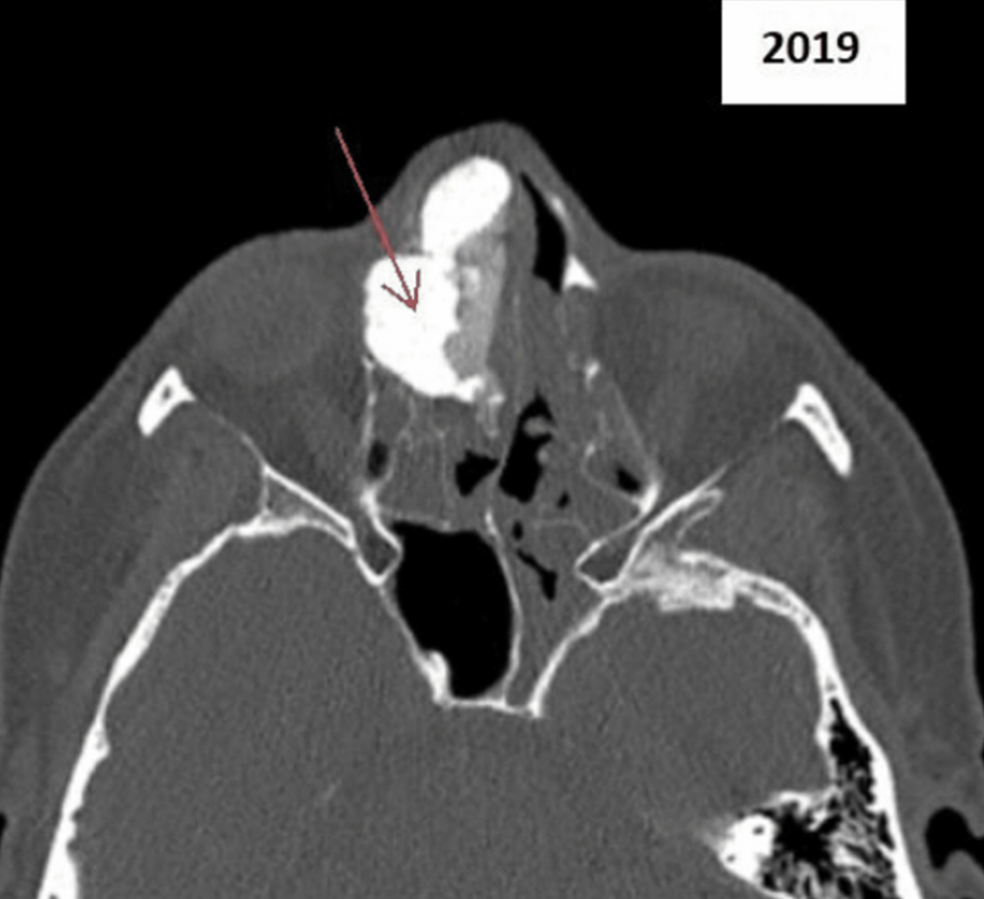
CT of sinuses in 2019 The red arrow is showing the osteoma.

**Figure 3:**
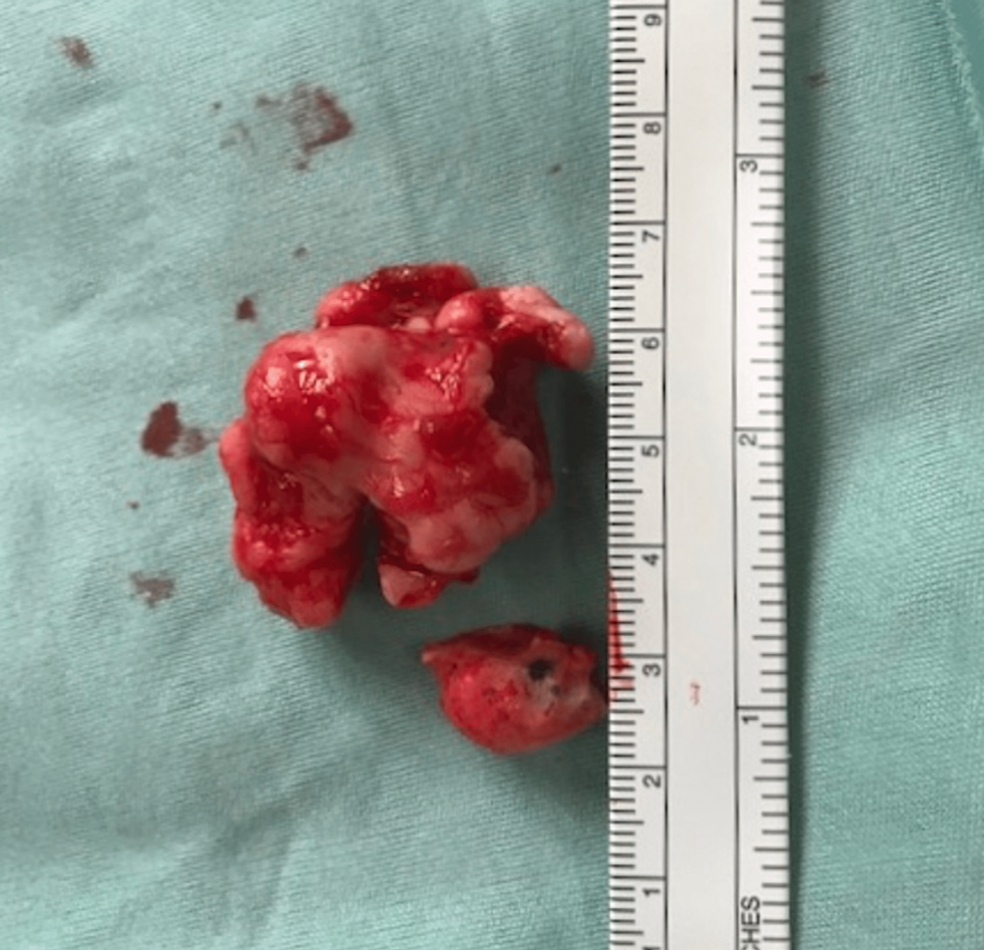
The osteoma after endoscopic removal

However, the patient re-presented to her GP 11 years later, in 2019, with a three-week history of new symptoms of unilateral nasal obstruction, proptosis, and epiphora. This was recognised as a "red-flag" presentation, and she was urgently referred to ENT under a suspected cancer two-week-wait. A new CT of the sinuses then revealed a large osteoma, which was splaying the nasal bone and causing restriction in her lateral gaze (Figure 2). It was deemed that the new presentation was due to "mass-effect" of the osteoma. Routine blood investigations (including full blood count, urea and electrolytes, and bone profile) were within normal ranges. Surgical intervention was the only treatment option, and via an endoscopic approach, the histologically confirmed osteoma was removed completely. It measured approximately 3cm (Figure 3). If it had not been surgically removed, it would have continued to grow and produce further obstructive, "mass-effect" symptoms. The patient’s post-operative period was uneventful [3].

## Discussion

Paranasal osteomas present insidiously due to their slow-growing nature. Key symptoms are related to “mass-effect” and are commonly nasal obstruction, facial pain, swelling, sinusitis, and nasal discharge. In rare cases, like ours, where significant growth has occurred, ophthalmological symptoms may also be present including diplopia, ptosis, and decreased visual acuity due to splaying of the nasal bone [1,2].

Gold-standard investigations include CT and magnetic resonance imaging (MRI) of the sinuses. It is thought that plain radiographic films are not sufficient [2]. Osteomas typically appear as homogenously calcified, lobulated and sharply defined tumours. Differentials for a suspected osteoma on CT include periosteal osteosarcoma, fibrous dysplasia, and osteoblastomas [1]. We believed the mass to be an osteoma (prior to histological confirmation) due to the relatively slow-growing history and the appearance on the CT scan as described above.

Osteomas that are not thought to be contributing to symptoms and have no “mass-effect” features do not commonly require immediate treatment. It has been estimated, based on a large case series, that the mean growth rate of paranasal sinus osteomas is 1.61mm per year; however, growth of up to 6.0mm per year has been reported in the literature [4]. Our case reminds clinicians that paranasal osteomas should have follow-ups using CTs to ensure that they are not growing and causing “mass-effect” on local structures [2]. At the time of writing this case report, there were no formal and established guidelines on the frequency and time-lapse of surveillance CT imaging. However, we recommend clinicians use their judgement based on clinical findings to make decisions on the frequency of CT imaging. Further data is needed to allow the production of formal guidelines.

Generally, surgical treatment of paranasal osteomas should be pursued if there are “mass-effect” symptoms, or if lesions occupy more than 50% of the sinus [2]. They are typically excised via an endoscopic approach or an open approach [1,2]. In our case, due to the location of the osteoma, an endoscopic approach was chosen.

## Conclusions

In conclusion, paranasal osteomas are rare benign bone tumours originating from the paranasal sinuses, often manifesting with non-specific symptoms and signs. At present, there is no formal guideline on how clinicians should approach surveillance imaging, however, the presented case report highlights the importance of being aware that osteomas can grow to affect local structures. Therefore, surveillance is necessary despite their slow-growing nature. Clinicians should use their clinical judgement and discretion on how to approach surveillance timings. Osteomas remain a rare presentation, and literature is scarce. Further data is needed to allow for future guidelines implementation.
